# Regional Gray Matter Volume Is Associated with Restrained Eating in Healthy Chinese Young Adults: Evidence from Voxel-Based Morphometry

**DOI:** 10.3389/fpsyg.2017.00443

**Published:** 2017-03-27

**Authors:** Yanhua Su, Todd Jackson, Dongtao Wei, Jiang Qiu, Hong Chen

**Affiliations:** ^1^Department of Psychology, Southwest UniversityChongqing, China; ^2^School of Management, Zunyi Medical UniversityZunyi, China; ^3^Department of Psychology, University of MacauTaipa, China

**Keywords:** restrained eating, regional gray matter volume, voxel-based morphometry, young adults, obesity

## Abstract

**Highlight**

- Participants were non-clinical young adults with different restrained eating levels.
- We assessed relations of restrained eating (RE) with regional gray matter volume (rGMV).
- High RE scores were related to larger GMV in specific areas related to reward.
- High RE scores were also linked to less GMV in regions related to response inhibition.

**Objective:** Dieting is a popular method of weight control. However, few dieters are able to maintain initial weight losses over an extended period of time. Why do most restrained dieters fail to lose weight? Alterations in brain structures associated with restrained eating (RE) represent one potentially important mechanism that contributes to difficulties in maintaining weight loss within this group. To evaluate this contention, we investigated associations between intentional, sustained restriction of food intake to lose or maintain body weight, and regional gray matter volume (rGMV) within a large non-clinical young adult, sample.

**Methods:** Participants (150 women, 108 men) completed measures of RE and demographics prior to undergoing an MRI scan. Voxel-based morphometry (VBM) evaluated strengths of association between RE scores and rGMV.

**Results:** Higher RE levels corresponded to more rGMV in regions linked to risk of overeating and binge-eating including the left insula and orbitofrontal cortex (OFC). Conversely, RE had significant negative correlations with rGMV in the left and right posterior cingulum gyrus, regions linked to inhibitory control and potential risk for future weight gain.

**Conclusions:** Together, findings suggested individual differences in RE among young adults correspond to GMV variability in regions linked to overweight and obesity risk.

## Introduction

Restrained eating (RE) or dietary restraint (DR), refers to the intentional, sustained restriction of food intake for purposes of weight loss or maintenance (Herman and Polivy, 1980). RE and DR have been assessed with several self-report measures including the Restraint Scale (RS; Herman and Polivy, 1980), Three Factor Eating Questionnaire (TFEQ; Stunkard and Messick, 1985) and Dutch Eating Behavior Questionnaire (DEBQ; Van Strien et al., 1986). Consistent with predictions of RE theories (Herman and Polivy, 1980; Heatherton et al., 1988), some evidence indicates women who score higher on these measures have significantly higher risk for future onset of binge eating, weight gain, and bulimic symptomatology compared to lower-scoring peers (Laessle et al., 1989; Johnson and Wardle, 2005; Stice et al., 2005). For example, Laessle (Laessle et al., 1989) found high RS scores were closely related to consequences of unsuccessful dieting including disinhibited eating and weight fluctuations, but were not associated with successful overall caloric restriction in everyday life. Other recent studies have also indicated REs are more prone to impulsivity, difficulties with response inhibition, and overeating (Jansen et al., 2009; Jasinska et al., 2012). Taken together, these studies indicate RE is typically difficult to maintain over extended intervals, especially in environments wherein cues for easily accessible, high-calorie foods abound.

Paralleling behavior differences, neuroimaging studies have corroborated differences between REs and unrestrained eaters (UREs) in the processing of food stimuli. For example, a positron emission tomography (PET) study indicated DEBQ DR subscale scores were positively correlated with dorsal striatum dopamine release during exposure to the smell and taste of food (Volkow et al., 2003). Coletta et al. (2009) used the RS to investigate neural activation differences between REs and UREs via functional magnetic resonance imaging (fMRI); they found REs showed more activation than UREs did in the left orbitofrontal cortex (OFC) and dorsolateral prefrontal cortex (DLPFC) in response to food pictures. Burger and Stice found higher RE scores based on the Dutch Restrained Eating Scale were related to hyper-responsivity in motivational and reward regions (right OFC, bilateral DLPFC) during food intake (Burger and Stice, 2011). Dong et al. (2014) used regional homogeneity (ReHo) analysis to assess resting-state activity among REs and UREs identified on the basis of RS scores. They found REs showed comparatively more ReHo in brain regions associated with food reward (i.e., OFC, DLPFC), attention (i.e., lingual gyrus, cuneus, inferior parietal lobule), and somatosensory functioning (i.e., paracentral lobule, anterior insula). DLPFC is known to be a high level cortex with multiple functions. One of the major function is the cognitive control (Greene et al., 2004; Egner and Hirsch, 2005). Nevertheless, as mentioned above, DLPFC has also shown to be a reward related region (Coletta et al., 2009; Burger and Stice, 2011; Dong et al., 2014).

Functional neuroimaging studies have shown food and food-related visual or olfactory cues can activate brain circuits implicated in reward such as the OFC and insula among REs. Whereas, the OFC is thought to encode information related to the reward value of food, the insula may contribute to processing information related to the taste of food and its hedonic value. Collectively, research implicating activation of these structures underscores how RE may strengthen the incentive salience of food cues, hence increasing risk for eating behavior in food-rich environments.

Behavior and fMRI correlates of RE have been documented, yet there is a relative paucity of research on associations between individual differences in RE and regional gray matter volume (rGMV), particularly within non-clinical samples. Voxel-based morphology (VBM) is an unbiased, whole-brain objective technique used to identify differences in the brain's gray matter (Ashburner and Friston, 2000). Gray matter comprises unmyelinated neurons and other cells distributed throughout the cerebral cortex, brainstem, cerebellum, and spinal cord. GMV is a product of cortical thickness and variations in surface area due to patterns of associated folds. Studies of brain anatomy can provide unique insights into the neural basis of dieting behavior. However, data are scarce in this regard. To our knowledge, only one study has investigated the association between eating behaviors and brain structural changes. Yao et al. (2016) employed VBM to examine the relationship between eating behavior traits, measured by TFEQ, and brain structural changes. They found cognitive restraint of eating was positively correlated with the GMV in the DLPFC and negatively correlated with the GMV in the putamen. As well, disinhibition scores were negatively associated with the GMV in the left middle frontal gyrus (Yao et al., 2016).

Yao et al.'s study is potentially instructive in establishing links between different aspects of eating behavior and alterations of various brain structures. However, it is not clear whether findings identified in that sample (age 20–60 years) apply to specific at risk groups such as young adults whose brains have not matured fully. Furthermore, despite considerable consistency in fMRI studies of RE, one possible source of variability in results may be in relation to the measurement of RE. Although popular RE scales share a common motivational component reflecting desire for thinness and concerns with shape and weight (Laessle et al., 1989), they diverge in other respects. For example, while high scores on salient TFEQ-2 and DEBQ subscales are said to tap successful dieting, the RS is a well-validated alternative on which high scores are more reflective of unsuccessful dieting (Laessle et al., 1989; Allison et al., 1992; Lowe, 1993; van Strien, 1999; Strien, 2002; van Strien et al., 2007; Stroebe et al., 2013). Finally, evaluating individual differences in RE and rGMV within non-clinical groups may clarify why people who score higher on RE measures have significantly higher risk for future onset of binge eating, weight gain, and bulimic symptomatology compared to lower-scoring peers. To address these issues, we assessed associations between RE and rGMV within a non-clinical sample of young women and men.

In addition, because RE may be multidimensional in nature, it is not clear whether some facets have stronger associations than others with rGMV. For example, the revised RS (Herman and Polivy, 1980) includes subscales that reflect concern for dieting (RS-CD) and weight fluctuations (RS-WF) that may be used in addition to total RS scores. Some authors have argued that RS-WF subscale items should be disregarded because they are potentially confounded with obesity and overweight (Stroebe, 2008). However, to explore these concerns empirically, links of each RS subscale and total RS scores with GMV can be assessed, after first statistically controlling for the impact of body mass index (BMI). Presumably, significant associations between RS-WF scores and GMV, independent of BMI, would suggest this subscale has utility beyond BMI.

Extending previous structural MRI research (Volkow et al., 2003), the objective of the current study was to examine structural changes associated with dietary restraint. In this study, we first used VBM to quantify GMV. Then, we conducted regression analyses to assess relations between GMV and total RS scores as well as e brain structural alterations associated with (I) concern for dieting (RS-CD) and (II) weight fluctuation dimensions of RS.

## Methods

### Participants and procedure

Participants were 258 right-handed, healthy undergraduate students (150 women, 108 men; mean age = 20.05, *SD* = 1.45, age range: 17–28 years; BMI = 20.30, *SD* = 1.96) from Southwest University (SWU) in Chongqing, China. The final sample was drawn from an initial sample of 297 students who completed an initial screen. However, 39 participants were not included because they did not complete the RS. Exclusion criteria also included self-reported current medical and/or psychiatric conditions including eating disorder diagnoses, a history of such conditions, and/or use of medication to treat current medical and/or psychiatric conditions.

The study was approved by the human research ethics committee at SWU. In accordance with the Declaration of Helsinki (World Medical Association., 1991), written informed consent was obtained from all participants prior to engaging in the study. Participants were recruited via an electronic bulletin board soliciting volunteers for research on associations between the brain and health. Participants were required to complete the RS and a demographics form prior to their MRI scan. In addition, objective assessment of height and weight was completed before the scan to permit calculation of body mass index (BMI) based on kg/m^2^.

Subsequently, participants underwent an MRI scan wherein they were instructed to keep their heads still and to remain awake. The scan was comprised of anatomical imaging (4 min, 18 s), resting state imaging (8 min, 8 s) and diffusion tensor imaging (17 min), but only anatomical imaging data was used in this study. On average, the study took 30 min to complete. Participants were compensated 80 RMB for completion of the research.

### Measures

Restraint Scale (RS; Herman and Polivy, 1980). The 10-item RS assesses dieting behavior, preoccupations with eating, binge eating behaviors, and past weight fluctuations. Exploratory factor analysis yielded a six-item concern with dieting (RS-CD) factor (e.g., “How often do you diet?”) and a four-item weight fluctuations (RS-WF) factor (e.g., “In a typical week, how much does your weight fluctuate?”). Items are rated on a five-point scale between *0* = *never* and *4* = *always*. Past research has supported the construct validity of the RS in Chinese samples (Kong et al., 2013). Alphas were α = 0.758 for the full RS, α = 0.636 for RS-CD, and α = 0.729 for RS-WF in the current sample. Full RS alphas were α = 0.640 for men, Alphas were α = 0.722 for women.

### MRI data acquisition and preprocessing

MR images were acquired with a 3.0-T Siemens Trio MRI scanner (Siemens Medical, Erlangen, Germany). High-resolution T1-weighted anatomical images were acquired using a magnetization-prepared rapid gradient echo (MPRAGE) sequence (repetition time (TR) = 1900 ms; echo time (TE) = 2.52 ms; inversion time (TI) = 900 ms; flip angle = 9°C; resolution matrix = 256 × 256; slices = 176; thickness = 1.0 mm; voxle size = 1 × 1 × 1 mm).

MR images were processed using SPM8 (Wellcome Department of Cognitive Neurology, London, UK; www.fil.ion.ucl.ac.uk/spm) implemented in Matlab 7.8 (MathWorks Inc., Natick, MA, USA). Each MR image was first displayed in SPM8 to screen for artifacts or gross anatomical abnormalities. For better registration, reorientation of images was manually set to the anterior commissure. Segmentation of images into gray matter and white matter was performed using new segmentation in SPM8. Subsequently, we performed Diffeomorphic Anatomical Registration through Exponentiated Lie (DARTEL) algebra in SPM8 for registration, normalization, and modulation (Ashburner, 2007). To ensure that regional differences in absolute GMV were conserved, the image intensity of each voxel was modulated by Jacobian determinants. Subsequently, registered images were transformed to Montreal Neurological Institute (MNI) space. Finally, normalized modulated images (gray matter and white matter images) were smoothed with an 8-mm full-width at half-maximum Gaussian kernel to increase the signal to noise ratio.

### Statistical analysis

Statistical analyses of GMV data were performed using SPM8. Following other published accounts (Ledberg et al., 1998; Takeuchi et al., 2014), in whole-brain analyses we used multiple linear regression analyses to identify regions in which regional gray matter volume (rGMV) was associated with RS scores. To control for possible confounding variables, BMI, age, gender and global GMV were entered as covariates within the regression model. We also applied explicit masking using the population-specific masking toolbox in SPM8 to restrict the search volume within gray matter and white matter (http://www.cs.ucl.ac.uk/staff/g.ridgway/masking/). In whole-brain analyses, multiple linear regressions were run to identify regions in which rGMV was associated with individual differences in RE. First, initial analyses were done and clusters were considered significant at the combined voxel-extent threshold of individual voxel levels (i.e., *P* < 0.001) and cluster extent >30 voxels, and the only significant correlation to emerge for RS scores was a positive association with GMV in a cluster that included left insula regions (BA 13). Second, there were some differences among the genders, sub-scales, to elucidate results further, the voxel-wise intensity threshold was set at *P* < 0.005 and a cluster-level threshold was calculated using the AlphaSim program in AFNI, with Monte Carlo simulation (Cox, 1996; Ward, 2000). AlphaSim is one method for multiple comparison corrections combining voxel intensity and cluster extent. Although the results corrected by Monte Carlo simulation might have some limitations reported in the study of Silver et al. (2011), it can provide the appropriate cluster-level threshold to achieve the desired false-positive rate (Liu et al., 2016). Effects were deemed to be significant when the volume of a cluster exceeded the minimum cluster size on whole brain GMV (determined using the Monte Carlo simulations; 683 voxels), in which case the probability of a type I error was < 0.05 (Silver et al., 2011). Finally, supplementary correlation analyses were performed within each gender to assess possible differences in patterns of association between RE and GMV (significance thresholds were set at *p* < 0.001, uncorrected).

## Results

### Sample characteristics

Table 1 provides sample means, standard deviations and ranges for age, BMI, and RS scores within the entire sample. As well, descriptive statistics for these measures are presented within subsamples of women and men. Tables 2–4 provide intercorrelations between demographics and RS scores for the entire sample and within each gender respectively. For the full sample and among the women, BMI had significant positive correlations with total RS, RS-CD, and RS-WF scores but was not significantly correlated with age (see Tables 2, 3). Among the men, BMI had significant positive correlations with age, total RS scores, and RS-WF scores but was not significantly correlated with RS-CD scores (Table 4).

**Table 1:** **Descriptive statistics of restrained eating sample (*N* = 258)**.

	**Total Sample (*n* = 258)**	**Range within sample**	**Men (*n* = 108)**	**Women (*n* = 150)**	***df***	***t***
Age	20.05 (1.45)	18–28	20.296 (1.42)	19.867 (1.459)	256	2.371
BMI	20.30 (1.96)	15.62–28.09	20.779 (2.17)	19.954 (1.72)	256	3.404^**^
RS (total)	9.11 (5.24)	0–25	6.426 (4.18)	11.007 (5.11)	256	−7.904^**^
RS-CD	5.18 (2.97)	0–17	3.972 (3.25)	6.007 (2.98)	256	−5.207^**^
RS-WF	3.93 (3.13)	0–12	2.454 (2.59)	5.000 (3.06)	256	−7.219^**^

*BMI, body mass index; RS, Restraint Scale (total); RS-CD, Restraint Scale-Concern with dieting subscale; RS-WF, Restraint Scale-Weight Fluctuations subscale*.

** *p < 0.01 (two-tailed)*.

**Table 2:** **Intercorrelations among the main research measures within the entire sample**.

	**1**	**2**	**3**	**4**
1. Age	−	−	−	−
2. BMI	0.178^**^		−	−
3. RS	−0.132^*^	0.248^**^	−	−
4. RS-CD	−0.128^*^	0.317^**^	0.815^**^	−
5. RS-WF	−0.062	0.339^**^	0.812^**^	0.478^**^

*BMI, body mass index; RS, Restraint Scale (total); RS-CD, Restraint Scale-Concern with dieting subscale; RS-WF, Restraint Scale-Weight Fluctuations subscale*.

* *p < 0.05 (two-tailed)*;

** p < 0.01 (two-tailed)

**Table 3:** **Intercorrelations among the main research measures for females**.

	**1**	**2**	**3**	**4**	**5**
1. Age	−				
2. BMI	−0.056	−			
3. RS	−0.146	0.447^**^	−		
4. RS-CD	−0.124	0.357^**^	0.841^**^	−	
5. RS-WF	−0.122	0.400^**^	0.851^**^	0.432^**^	−

*BMI, body mass index; RS, Restraint Scale (total); RS-CD, Restraint Scale-Concern with dieting subscale; RS-WF, Restraint Scale-Weight Fluctuations subscale*.

** *p < 0.01 (two-tailed)*.

**Table 4:** **Intercorrelations among the main research measures for males**.

	**1**	**2**	**3**	**4**	**5**
1. Age	−				
2. BMI	0.359^**^	−			
3. RS	0.059	0.353^**^	−		
4. RS-CD	−0.073	−0.013	0.786^**^	−	
5. RS-WF	0.188	0.587^**^	0.627^**^	0.012	−

*BMI, body mass index; RS, Restraint Scale (total); RS-CD, Restraint Scale-Concern with dieting subscale; RS-WF, Restraint Scale-Weight Fluctuations subscale*.

** *p < 0.01 (two-tailed)*.

### Correlations between RS scale scores and rGMV

**Figure 1:**
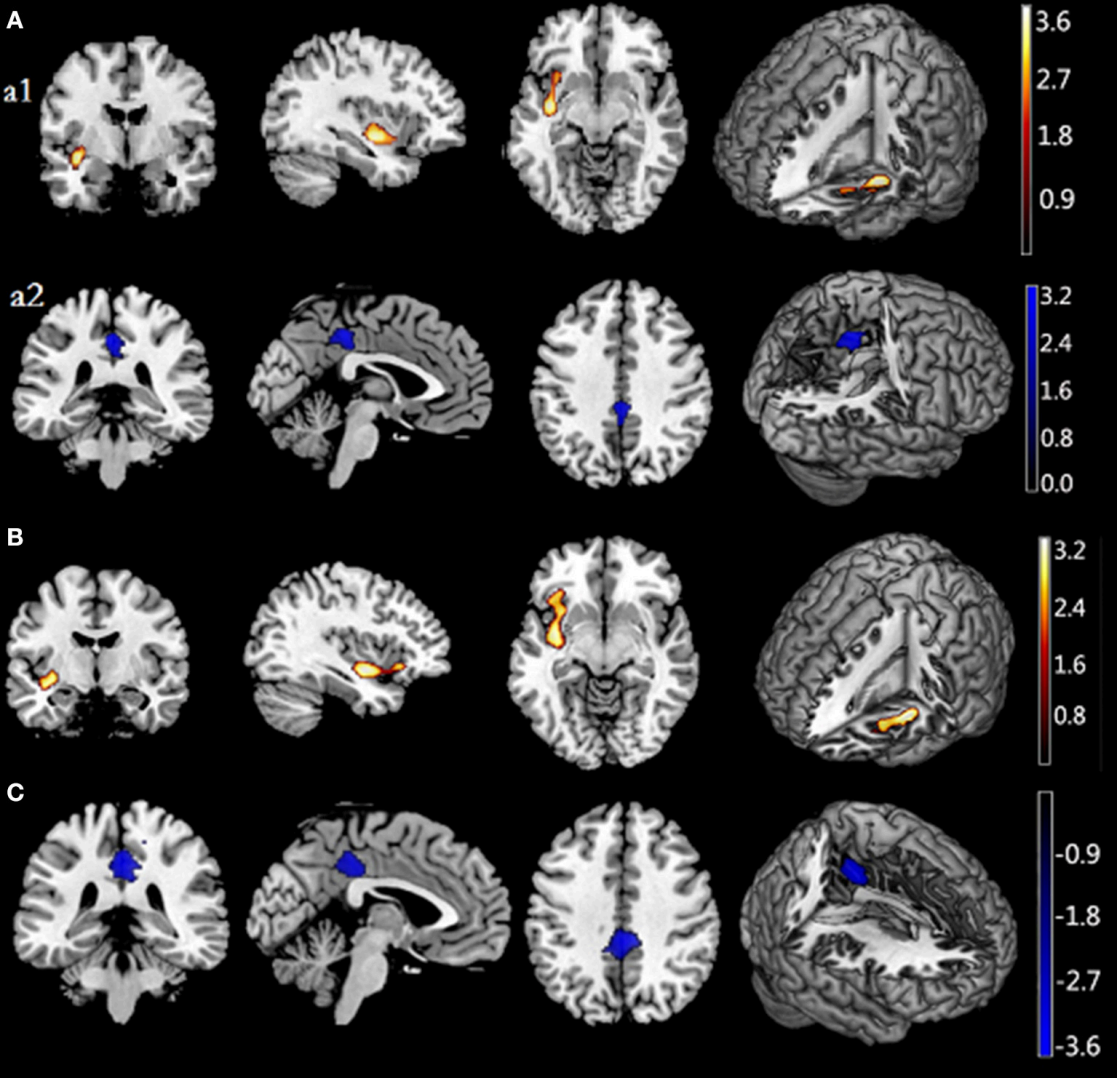
**Associations of Restraint Scale (RS) total and subscale scores with regional gray matter volume**. **(A)** shows a positive association between rGMV and RS scores in the left insula **(a1)** and a negative association between rGMV and RS scores in the posterior cingulum gyrus **(a2)**. **(B)** shows positive correlations between RS-CD scores and GMV in the left insula, extending to the orbitfrontal cortex. **(C)** shows a negative association between rGMV and RS-WF in the posterior cingulum gyrus. Significant regions are shown in red-yellow (positive) and black-blue (negative). RS, Restraint Scale (total); RS-CD, Restraint Scale-Concern with dieting subscale; RS-WF, Restraint Scale-Weight Fluctuations subscale; Rgmv, regional gray matter volume.

After entering age, sex, BMI and global GMVs as covariates, a multiple regression analysis within the entire sample indicated total RS scores had significant, positive correlations with GMV in a cluster that included left insula areas. Total RS scores were also negatively correlated with GMV in a cluster that included the posterior cingulum gyrus (see Figure 1, Table 5).

**Table 5:** **Associations of restraint scale scores, concern with dieting, and weight fluctuation scores with gray matter volume**.

	**Brain region**	**Hemisphere**	**MNI coordinates**			**Voxels size**	**Peak *T*-value**	**Correlation coefficient**
			**x**	**y**	**z**			
Total RS	Left insula (BA 13)	L	−38	−6	−9	1025	3.68	0.20^**^
	Posterior cingulum gyrus (BA 21)		0	−35	42	510	−3.52	−0.23^**^
RS-CD	Left insula/ orbitofrontal cortex (BA 12, 13)	L	−38	−3	−11	915	3.41	0.22^**^
RS-WF	Posterior cingulum gyrus (BA 21)		0	−35	41	1269	−3.58	−0.30^**^

*RS, Restraint Scale (total); RS-CD, Restraint Scale-Concern with dieting subscale; RS-WF, Restraint Scale-Weight Fluctuations subscale*.

*Results are significant at P < 0.05, corrected for multiple comparisons at a cluster level with non-stationary corrections and an underlying voxel level of P < 0.005, corrected*.

** *p < 0.01 (two-tailed)*.

**Figure 2:**
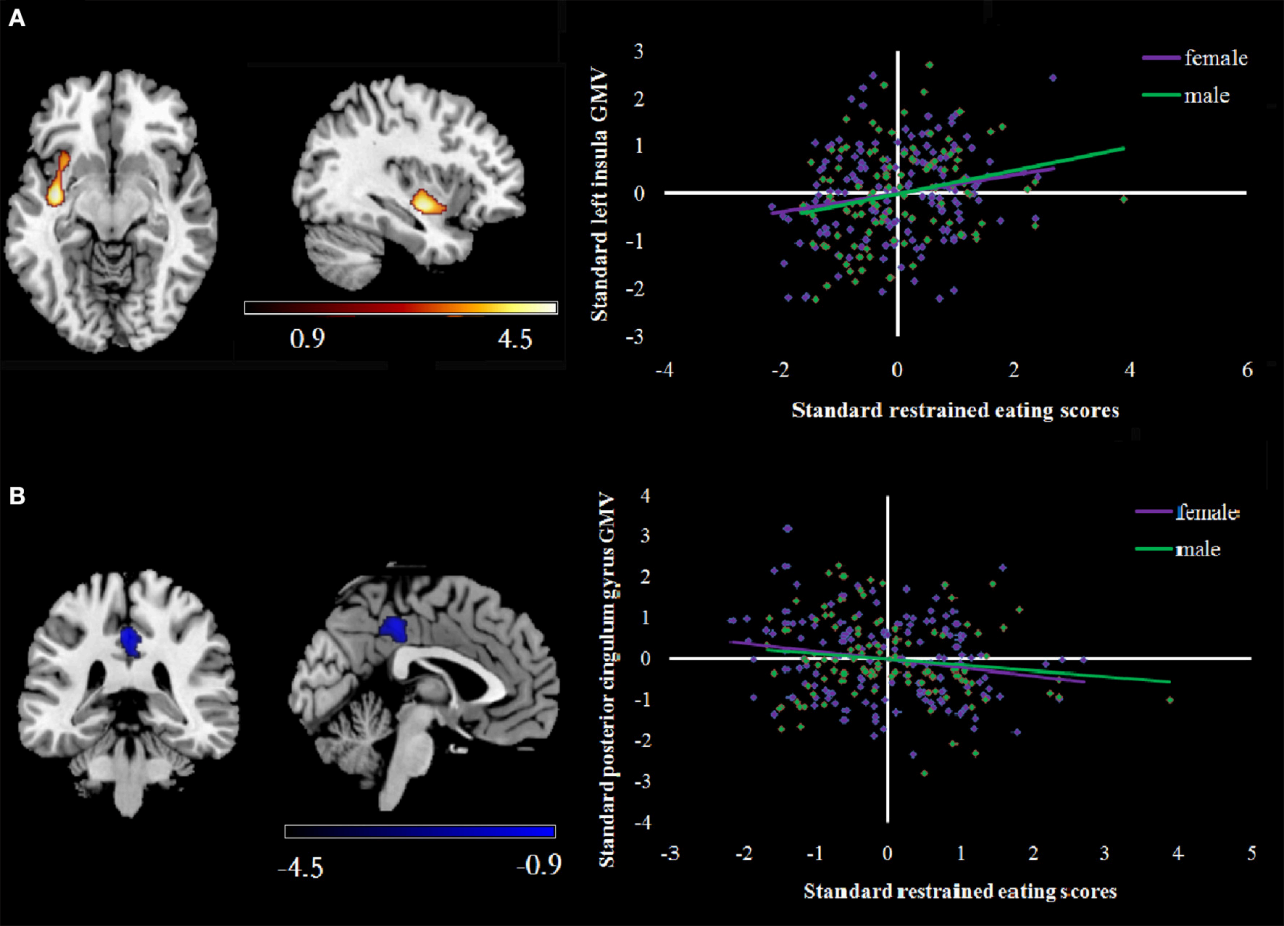
**(A)** shows a scatterplot of positive correlations between rGMV and RS scores in the left insula. rGMV and RS were normalized and adjusted for age, sex, and total brain volume within each gender. **(B)** shows a scatterplot of the negative correlations between rGMV and RS scores in the posterior cingulum gyrus within each gender. Correlations are shown in purple (female) and green (male). rGMV, regional gray matter volume.

To elucidate results further, we assessed relations of RS subscale scores with GMV in the entire sample. The only significant correlation to emerge for RS-CD scores was a positive association with GMV in a cluster that included left insula regions (BA 13), extending to the orbitofrontal cortex (BA 12). Conversely, RS-WF scores were negatively correlated with GMV in the posterior cingulum gyrus (see Figures 1, 2, Table 5).

Supplementary analyses also evaluated associations between RS scores and GMV within each gender. Among the women, after entering age, BMI and global GMVs as covariates, a multiple regression analysis indicated total RS scores had significant, positive correlations with GMV in a cluster that included left insula, middle orbitofrontal cortex and parahippocampal gyrus. In addition, total RS scores were negatively correlated with middle frontal gyrus and posterior cingulum gyrus GMV. For men, total RS scores had positive correlations with GMV in the left insula and middle temporal gyrus in addition to a negative correlation with GMV in the posterior cingulum gyrus (see Table 6).

**Table 6:** **Associations of restraint scale total with gray matter volume within each gender**.

	**Brain region**	**Hemisphere**	**MNI Coordinates**			**Voxels**	**Peak *T*-value**	**Correlation coefficient**
			**X**	**y**	**z**	**Size**		
Women	Left insula (BA 13)	L	−38	−6	−9	1025	3.99	0.20^**^
	Middle orbitofrontal cortex (BA 12)	L	−26	32	−14	251	3.85	0.25^*^
	Parahippocampal gyrus	R	25	−26	−18	49	3.56	0.21^*^
	Posterior cingulum gyrus (BA 21)		0	−35	42	142	−3.52	−0.32^**^
	Middle frontal gyrus (BA 46)	L	−42	22	44	34	−3.67	−0.19^*^
Men	Left insula (BA 13)	L	−38	−3	−11	75	3.4	0.25^**^
	Middle temporal gyrus (BA 21)	L	−26	−12	−7	42	3.31	0.23^*^
	Posterior cingulum gyrus (BA 21)		0	−34	41	32	−3.84	−0.14^*^

*RS, Restraint Scale (total). Results are significant at P < 0.001, uncorrected*.

* *p < 0.05 (two-tailed)*;

** *p < 0.01 (two-tailed)*.

Direct comparisons between correlations within subgroups of women and men were performed. There was no difference between correlations of women vs. men in the left insula (*p* = 0.68), or posterior cingulum gyrus (*p* = 0.14).

## Discussion

To the best of our knowledge, this study is the first to use VBM analyses to investigate associations between RE and rGMV in a large, non-clinical sample of women and men. Results were in line with the general hypothesis that individual differences in overall RS scores would correspond to significant structural differences in brain regions associated with reward and inhibition.

First, within the entire sample, high total RS scores were associated with larger GMV in the left insula (BA 13), an area implicated in processing interoceptive information reflecting hunger, thirst, and visceral sensations (Craig, 2011). The insula, striatum and midbrain structures encode the subjective value of rewards regardless of their type (Sescousse et al., 2010). Moreover, increased insula GMV in eating disorder groups relative to non-disordered controls is associated with taste pleasantness and reward value. Smucny and colleagues found GMV was lower in the insula, medial OFC, and cerebellum in obese-prone compared to obese-resistant individuals, suggestive of structural differences in brain regions important in energy regulation among persons at risk for weight gain (Smucny et al., 2012). Functional neuroimaging studies have linked stronger insula activation to encoding food liking (Volkow et al., 2011) and exposure to appetizing food pictures among REs (Coletta et al., 2009). Other research has found REs are more likely than UREs to show heightened reward circuitry in response to food cues that increase risk for overeating (Jasinska et al., 2012). However, Yao et al. (2016) found cognitive restraint of eating was positively correlated with DLPFC GMV and negatively correlated with GMV in the putamen. We may conclude that the inconsistent results are probably due to differences between the various restraint scales (Yao et al., 2016).

In the context of most other studies, our finding of an increased GMV in the insula implies REs may showy an enhanced drive for compulsive eating that interferes with the control of eating, in turn, increasing risk for obesity. However, because there is unlikely to be a one-to-one correspondence between fMRI activation and GMV in specific regions, future studies that evaluate associations between RE, insular rGMV and BOLD signal changes in response to food stimuli are needed to elucidate these associations further.

Total RS scores were also inversely correlated with GMV in the posterior cingulum gyrus (CGp). Several MRI studies have suggested the CGp has a more fundamental role in the allocation of neural resources to cognitive control akin to that of attention in the selective processing of sensory stimuli (Maddock et al., 2001, 2003; Greicius et al., 2004; Luhmann et al., 2008). In food-rich environments, the long-term goal of weight control as a self-regulation process requires more cognitive resources than the proximal goal of food enjoyment does. There is evidence that both (i) proactive self-control efforts in response to immediate temptations and (ii) externally-imposed controls can help to protect long-term interests such as losing or maintaining weight (Takeuchi et al., 2014). However, it is plausible that weak self-control efforts and/or lack of responsiveness to external controls in response to immediate temptations impair one's ability to monitor or inhibit inappropriate behavior, consequently disrupting long-term weight control goals. An associated hypothesis that warrants attention within future longitudinal extensions is the possibility that more rGMV in food reward regions and/or less rGMV in the CGp undermines self-control capacities of REs in response to enticements, in turn, increasing vulnerability to future overeating and weight gain.

In disentangling features of RE further, higher RS-CD subscale scores were correlated with increased GMV in a cluster that included left insula areas extending to the orbitofrontal cortex (OFC). The insula (BA 13) and OFC (BA 12) comprise areas in a network of regions involved in processing food stimuli, emotion, awareness of internal state, and behavioral inhibition, and are believed to integrate sensory information that affects eating and reward-related behavior (Craig, 2010; Burger and Stice, 2011; Weise et al., 2013). Also, Cristofori et al. (2015) pointed out that connections from the insula to the OFC could allow the insula to integrate the value of rewards in modulating internal states (Cristofori et al., 2015). In another VBM study, Schäfer et al. (2010) found that, compared to non-eating disordered controls, both bulimia nervosa and binge-eating disorder groups were characterized by increased medial OFC GMV reflecting possible group differences in food reward processing. Neuroimaging studies have also supported medial OFC involvement in subjective evaluations of reward (Kringelbach et al., 2003) and activation during the anticipation and experience of pleasant tastes (O'Doherty et al., 2002; Kringelbach, 2005). Another recent study found that high restraint scores of adolescent girls were related to more right OFC activation in response to the taste of small amounts of milkshake (Burger and Stice, 2011). In the context of other VBM and fMRI studies, the present findings suggested high levels of concern with dieting correspond to larger GMV in food reward regions.

RS-WF subscale scores in the sample were negatively correlated with GMV in the posterior cingulum gyrus (CGp), partially in line with Brooks et al. (2011) who observed reduced right posterior cingulate GMV among women with AN, a group preoccupied with weight changes, relative to non-disordered controls. Past fMRI research has implicated CGp involvement in allocating neural resources toward cognitive control (Maddock et al., 2003). Presumably, when attempts to control food intake among REs are accompanied by reduced cognitive control or impaired self-regulation, vulnerability to overeating and weight changes increase (Vohs and Heatherton, 2000). In light of the negative relation of RS-WF scores with GMV in this sample, prospective studies that evaluate (i) relations between reported weight fluctuations and changes in CGp GMV volumes as well as (ii) complementary associations between CGp GMV and changes in BMI or perceived weight fluctuations can clarify implications of this correlation further.

Finally, within each gender, associations between RE and GMV in the left insula and posterior cingulum gyrus replicated those observed for the entire sample. However, among the women, RE also had positive and negative correlations with GMV, respectively, in the parahippocampal gyrus and middle frontal gyrus (MFG). The parahippocompal gyrus may have an key role in memory encoding and retrieval. However, of more possible relevance here, inhibition of inappropriate responses, control of goal-directed behaviors, and ability of error detection/correction are key MFG functions (Menon et al., 2001). Yokum et al. found trend-level reductions in MFG GMV were related to BMI increases over a 1-year follow-up (Yokum et al., 2012). In concert with that study, a plausible hypothesis that follows from the present finding is that lower GMV in the MFG among women with higher RE levels may correspond to reduced inhibitory control that increases vulnerability to overeating and weight gain in food-rich environments. Among the men, RE scores had a unique significant positive correlation with middle temporal gyrus GMV, consistent with hypothesized links between RE and GMV in food reward areas. Specifically, these data converge with Dong et al. (2014) who observed positive correlations between RE and resting state activity in the MTG and other regions involved in visual perception and food reward.

### Limitations, future research directions, and conclusions

Despite its possible implications, three limitations of this study, in particular, must be elaborated. First, participants were highly educated young adults so findings may not generalize to other segments of the general population or samples with significant clinical eating disturbances. Extensions to more varied samples might elucidate general changes in GMV associated with RE and those related to specific developmental windows. Second, although use of a cross-sectional design was a sensible initial approach to examining structural correlates of RE, as alluded to above, the status of RE and rGMV as causes or risk factors for changes in one another cannot be determined with this approach. In light of bivariate associations observed in this study and conceptually similar research (Jasinska et al., 2012), longitudinal extensions would help to clarify the potential interplay between RE and subtle changes in rGMV over time. Third, select differences in GMV correlates of RE between women and men, underscore the need for future studies that comprise both genders rather than relying exclusively on data from women to understand GMV correlates of unsuccessful RE.

In summary, this exploratory study suggested individual differences in RS are related to variability in rGMV among young Chinese adults. Within each gender, unique RE-GMV associations were observed with regions linked to food reward and/or inhibition. Results also point to future work assessing the hypothesis that changes in brain structures such as the insula and posterior cingulum gyrus underlie difficulties in maintaining weight loss and risk of overeating.

## Ethics statement

This study was carried out in accordance with the recommendations of the guidelines for human research, the human research ethics committee at SWU with written informed consent from all subjects. All subjects gave written informed consent in accordance with the Declaration of Helsinki. The protocol was approved by the the human research ethics committee at SWU.

## Author contributions

JQ and HC design the study; YS and DW collect the data; YS analyzes and interprets the data; YS and TJ write the manuscript.

### Conflict of interest statement

The authors declare that the research was conducted in the absence of any commercial or financial relationships that could be construed as a potential conflict of interest.
